# Preschool Oscillometry and Later Asthma-Related Outcomes: A Systematic Review of Longitudinal Studies

**DOI:** 10.3390/children13060756

**Published:** 2026-05-29

**Authors:** Dafni Moriki, Dimosthenis Maris, Aspasia Mavronasou, Panagiotis Dalamarinis, Despoina Koumpagioti, Maria Tsouprou, Vasilis Grammeniatis, Michalis Kalogiannis, Angeliki Galani, Eleni A. Kortianou, Konstantinos Douros

**Affiliations:** 1 Pediatric Allergy and Respiratory Unit, 3rd Department of Pediatrics, “Attikon” University Hospital, School of Medicine, National and Kapodistrian University of Athens, 12462 Athens, Greece; dmoriki@med.uoa.gr (D.M.); pdalamarinis@med.uoa.gr (P.D.); mariatsoup@gmail.com (M.T.); mixalis_kalogiannis11@hotmail.com (M.K.); anggalani@med.uoa.gr (A.G.); 2 1st Department of Pediatrics, “Agia Sofia” Children’s Hospital, 11527 Athens, Greece; dimosmaris@med.uoa.gr; 3 Clinical Exercise Physiology & Rehabilitation Research Laboratory, Physiotherapy Department, School of Health Sciences, University of Thessaly, 35132 Lamia, Greece; asmavronasou@uth.gr (A.M.); ekortianou@uth.gr (E.A.K.); 4 Department of Nursing, University of West Attica, 12243 Athens, Greece; dkoumpagioti@uniwa.gr; 5 Department of Pediatrics, 1st Pediatric Clinic, Agia Sofia Hospital, 11527 Athens, Greece; 6 Pediatric Department, General Hospital of Ioannina G. Hatzikosta, 45445 Ioannina, Greece; bilgramm@gmail.com

**Keywords:** oscillometry, impulse oscillometry, preschool wheezing, asthma risk, lung function

## Abstract

**Highlights:**

**What are the main findings?**

- Abnormal preschool oscillometry, particularly elevated resistance at 5 Hz (R5), was consistently associated with later asthma-related outcomes across longitudinal studies.
- Early oscillometric abnormalities were also linked to lower later lung function, including reduced FEV_1_ and FEV_1_/FVC, suggesting persistent airflow limitation into later childhood and adolescence.

**What are the implications of the main findings?**

- Preschool oscillometry may provide useful complementary prognostic information for identifying children at higher risk of persistent asthma and later lung function impairment.
- Current evidence is too limited and heterogeneous to support a single validated cutoff or routine stand-alone clinical use, underscoring the need for larger standardized prospective studies.

**Abstract:**

**Background:** Preschool wheezing is common, but distinguishing transient wheezing from early persistent asthma remains difficult. Oscillometric techniques, including impulse oscillometry (IOS), may provide objective prognostic information. **Objective:** To systematically review longitudinal studies assessing whether oscillometric measurements obtained at preschool age are associated with later asthma-related outcomes or lung function impairment. Methods: PubMed, Scopus, and MEDLINE (via EBSCOhost) were searched from inception to 28 February 2026. Eligible studies included preschool-aged children or closely related early-childhood populations assessed with IOS or forced oscillation technique (FOT), longitudinal follow-up, and later asthma-related or spirometric outcomes. Two reviewers independently screened studies, extracted data, and assessed risk of bias with QUIPS. Owing to substantial heterogeneity, findings were synthesized qualitatively. **Results:** Seven longitudinal cohort studies, encompassing 1077 children, met the eligibility criteria; baseline oscillometry was performed between ages 2 and 7 years, and follow-up ranged from 1 to 10 years. Short-term classification and longer-term prognostic studies were interpreted separately. Resistance-based indices, particularly R5, showed the most consistent associations with later asthma-related outcomes and lower subsequent spirometric indices, including FEV_1_ and FEV_1/_FVC. Most studies were at moderate risk of bias, and some reports came from related cohorts. **Conclusions:** Preschool oscillometry may provide complementary physiological information to assess asthma risk in symptomatic or high-risk preschool-aged children, particularly through resistance-based measures. However, evidence remains limited, heterogeneous, and insufficient to support a single validated cutoff, routine screening of apparently healthy children, or routine stand-alone prognostic use.

## 1. Introduction

Asthma is one of the most prevalent chronic respiratory diseases in childhood and represents a major global health burden [1]. Epidemiological data indicate that a substantial proportion of all asthma cases begin before the age of six [2,3]. Wheezing is common in preschool-aged children and is often transient, particularly when associated with viral respiratory infections [4,5]. However, some children develop persistent asthma, characterized by chronic airway inflammation, bronchial hyperresponsiveness, and variable airflow obstruction [6,7,8]. Distinguishing transient wheezing from early persistent asthma remains a major challenge in pediatric respiratory medicine [5,9].

Early identification of children at increased risk of persistent asthma is clinically important, as delayed diagnosis and treatment are associated with greater symptom burden, more frequent exacerbations, reduced lung function, and increased healthcare utilization [10,11]. Clinical predictors such as recurrent wheezing, atopy, eczema, and family history are informative, but prediction models have shown only moderate performance and limited external generalizability [12,13,14]. Objective assessment of lung function may, therefore, improve early risk stratification [15]. In preschool-aged children, however, spirometry is often limited by the need for cooperation and forced expiratory maneuvers [16,17,18,19,20].

Oscillometry assesses respiratory mechanics by applying small pressure oscillations at the mouth during quiet tidal breathing and analyzing the pressure–flow relationship [21,22]. Forced oscillation technique (FOT) refers to the original methodological principle, whereas impulse oscillometry (IOS) is a commercially developed form of FOT that uses brief pressure impulses containing a range of frequencies [21,22,23]. Thus, IOS is an implementation of the broader forced oscillation approach. Both methods estimate respiratory system impedance, although values may vary according to device, signal type, quality-control procedures, reference equations, and reporting conventions [21,22].

Respiratory impedance includes resistance and reactance. Resistance reflects the frictional opposition to airflow within the airways, whereas reactance reflects the elastic and inertial properties of the respiratory system [21]. This makes oscillometry attractive in preschool-aged children as it is non-invasive, effort-independent, and can often be performed with minimal active cooperation [16,21].

The most commonly reported resistance parameters are R5 and R20. R5, resistance at 5 Hz, reflects total respiratory system resistance, including central and peripheral airway contributions. R20, resistance at 20 Hz, is relatively more influenced by central or proximal airway resistance, although neither measure is anatomically specific. Higher R5 or R20 generally indicates increased airway narrowing or obstruction. R5-20, the difference between R5 and R20, summarizes the frequency dependence of resistance and is often used as an indirect, nonspecific signal of ventilation heterogeneity or possible peripheral airway involvement. Increased R5-20 values are generally considered unfavorable [23,24,25,26]. The slope of resistance across frequency, dRrs/df, is less routinely used and describes how resistance changes with oscillation frequency. A steeper negative slope, or a lower dRrs/df, depending on the reporting convention, may reflect heterogeneous respiratory mechanics, but it is less standardized and less clinically established than R5 or R5-20 [21,24].

Reactance parameters provide complementary information. X5, reactance at 5 Hz, is usually negative in young children, and more negative values may suggest reduced compliance, altered elastic properties, ventilation heterogeneity, or possible peripheral airway obstruction [21,22,23,24]. AX, the area of reactance, summarizes the cumulative reactance abnormality between 5 Hz and the resonant frequency, while Fres is the frequency at which reactance crosses zero. Higher AX and Fres generally indicate greater mechanical abnormality, but these indices are not anatomically specific and should be interpreted in a clinical context, with attention to age, lung volume, technical quality, reference equations, and reporting conventions [21,23,27]. A practical summary of commonly reported oscillometry indices and their clinical interpretation is provided in Table 1.

Among oscillometric parameters, R5 is the most widely reported and currently the best-established individual marker in pediatric clinical and research settings. R5-20, X5, AX, Fres, and dRrs/df may provide additional information on frequency-dependent or reactance-related mechanics, but their thresholds and prognostic interpretation are less uniform across studies [23,24,25,26,27]. Over the past two decades, longitudinal studies have examined whether abnormal oscillometry in early childhood predicts later asthma and impaired lung function [28,29,30,31,32,33,34]. However, interpretation remains difficult because studies differ in populations, oscillometric indices, bronchodilator status, thresholds, follow-up duration, outcome definitions, technical quality, and cohort independence.

Accordingly, this study systematically reviews longitudinal evidence on whether preschool-age or closely related early-childhood oscillometric measurements are associated with later asthma-related outcomes and long-term lung function impairment.

## 2. Materials and Methods

### 2.1. Literature Search Strategy

This systematic review followed the Preferred Reporting Items for Systematic Reviews and Meta-Analyses (PRISMA) 2020 guidelines [35] and was prospectively registered in the International Prospective Register of Systematic Reviews (PROSPERO) (CRD420261296620). The registered protocol was initially specified as a systematic review and meta-analysis. After study selection and data extraction, quantitative synthesis was judged inappropriate because the eligible studies were few in number and showed substantial clinical and methodological heterogeneity, including differences in study populations, potential overlap between some cohorts, oscillometric indices and their expression, bronchodilator status, follow-up duration, and outcome definitions. Accordingly, the review was completed as a qualitative systematic review of prognostic longitudinal evidence rather than a meta-analysis (Supplementary Table S1).

The review question was structured according to the PICOTS framework shown in Table 2. PubMed, Scopus, and MEDLINE (via EBSCOhost) were searched from inception to 28 February 2026. The search strategy combined controlled vocabulary terms and free-text keywords related to preschool children, oscillometry, wheezing, and asthma outcomes. Searches were limited to studies published in English. Reference lists of relevant articles and reviews were also manually screened to identify additional eligible studies. The detailed database-specific search strategies are provided in Appendix A.

### 2.2. Inclusion and Exclusion Criteria

Eligibility criteria were defined a priori according to the PICOTS framework presented in Table 2. Studies were eligible if they included children undergoing oscillometric assessment during preschool or closely related early-childhood ages and reported longitudinal asthma-related outcomes or subsequent lung function impairment. To avoid excluding otherwise relevant cohorts, studies in which measurements were obtained slightly beyond 6 years of age were also considered eligible, provided that the population and clinical question remained focused on preschool wheezing and the risk of later asthma. Future asthma was defined as physician-diagnosed asthma, active asthma at follow-up based on symptoms and/or medication use, or asthma diagnosis according to validated criteria. Secondary outcomes included long-term impairment in lung function, such as reduced forced expiratory volume in one second (FEV_1_), reduced ratio of forced expiratory volume in one second to forced vital capacity (FEV_1_/FVC), or persistent airflow obstruction.

Studies were excluded if they were cross-sectional and lacked longitudinal follow-up, focused solely on asthma control or exacerbations without assessing subsequent asthma-related outcomes, did not include oscillometric assessment during the preschool period, or were non-original research articles, including case reports, narrative reviews, editorials, or conference abstracts without full data.

### 2.3. Study Selection and Data Extraction

Two reviewers independently screened titles and abstracts and subsequently assessed the full texts of potentially eligible studies. Disagreements were resolved through discussion or consultation with a third reviewer. Where multiple publications arose from overlapping cohorts, all relevant reports were retained for outcome-specific qualitative synthesis, but overlap was explicitly considered when interpreting the apparent consistency of findings. The study selection process was documented using a PRISMA flow diagram.

Data extraction was also performed independently by two reviewers using a standardized form. Extracted data included study characteristics (author, year, country, design, and sample size), participant characteristics, oscillometry methods and reported parameters, outcome definitions, follow-up duration, and effect estimates. Adjusted estimates were preferentially extracted where available.

### 2.4. Risk of Bias Assessment

Risk of bias was assessed using the Quality In Prognosis Studies (QUIPS) tool, which assesses six domains: study participation, study attrition, prognostic factor measurement, outcome measurement, study confounding, and statistical analysis and reporting. All assessments were performed independently by two reviewers, and disagreements were resolved through discussion.

### 2.5. Data Synthesis

A qualitative synthesis was conducted for all included studies and structured according to the main outcomes of interest, namely later asthma-related outcomes and longitudinal lung function impairment. Oscillometric parameters were categorized by physiological domain, including resistance-based measures (R5, R20), frequency-dependent resistance indices (R5-20 and dRrs/df), reactance-based measures (X5), and reactance-derived indices (AX and Fres). These categories were used descriptively and were not assumed to represent anatomically specific airway compartments. Effect estimates were extracted as reported by each study, including odds ratios (ORs), regression coefficients, correlation coefficients, sensitivity/specificity values, and area-under-the-curve (AUC) metrics, and adjusted estimates were preferentially summarized when available. Due to heterogeneity in oscillometric parameter expression (absolute values, percent predicted, or z-scores), bronchodilator status, outcome definitions, follow-up duration, threshold definitions, and partial cohort overlap, results were summarized descriptively rather than pooled quantitatively. Because there were too few studies for meaningful small-study-effect analyses and no meta-analysis was performed, no formal reporting-bias assessment was undertaken.

### 2.6. Certainty of Evidence Assessment

Because the included evidence was limited and heterogeneous, and no meta-analysis was performed, certainty of evidence was assessed narratively for the two main outcome domains (later asthma-related outcomes and later lung function impairment). Judgments were based on study limitations/risk of bias, consistency of direction of findings, precision of estimates, heterogeneity of methods and outcomes, and the presence of overlapping cohorts. In keeping with the prognostic and predominantly observational nature of the evidence base, and because a pooled estimate was not generated, certainty was expressed qualitatively rather than with a formal GRADE profile.

## 3. Results

### 3.1. Study Selection

**Figure 1:**
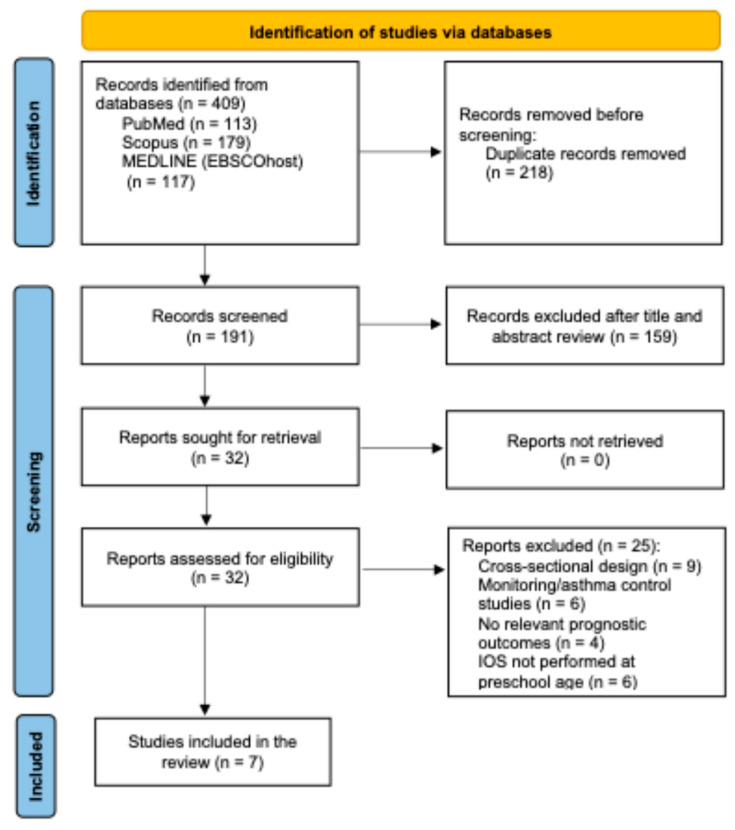
PRISMA 2020 flow diagram of the study selection process.

The database search yielded 409 records. After removal of duplicates, 191 records were screened by title and abstract, of which 32 underwent full-text review. Seven longitudinal studies met the predefined criteria and were included in the qualitative synthesis. The study selection process is summarized in Figure 1.

### 3.2. Study Characteristics

The qualitative synthesis included seven longitudinal cohort studies involving 1077 children. Baseline oscillometry was performed between 2 and 7 years of age. Specifically, children were assessed at ages 2–7 years in Knihtilä 2015 [33], 3–6 years in Chen 2024 [31], 3–5 years in Grell 2023 [29], 3–7 years in Lajunen 2020 [32], 4–7 years in Lajunen 2018 [30], 5–7 years in Lauhkonen 2018 [34], and serially at ages 4, 5, and 6 years in Knihtilä 2024 [28]. Follow-up duration ranged from 1 to 10 years, with final sample sizes ranging from 64 to 219 participants across studies, reflecting variable attrition. Outcome assessment ranged from early school age, approximately 4–7 years after short-term follow-up, to adolescence at 12–18 years of age. Study populations included children with recurrent wheezing, asthma-like symptoms, persistent asthma, or children from high-risk cohorts. Therefore, the available evidence mainly reflects clinically symptomatic or high-risk preschool-aged children, rather than apparently healthy, unselected populations. Later asthma-related outcomes were assessed in four studies [28,30,31,32], while longitudinal spirometric outcomes were assessed in six studies [28,29,30,32,33,34]. All studies used IOS. Potential cohort overlap was considered explicitly: Lajunen 2018 [30] and Lajunen 2020 [32] appear to derive from the same Helsinki longitudinal asthma cohort, Knihtilä 2015 [33] was considered institutionally related without clearly documented overlap, and Lauhkonen 2018 [34] was separate. The characteristics of the included studies are presented in Table 3.

### 3.3. Risk of Bias Results

**Figure 2:**
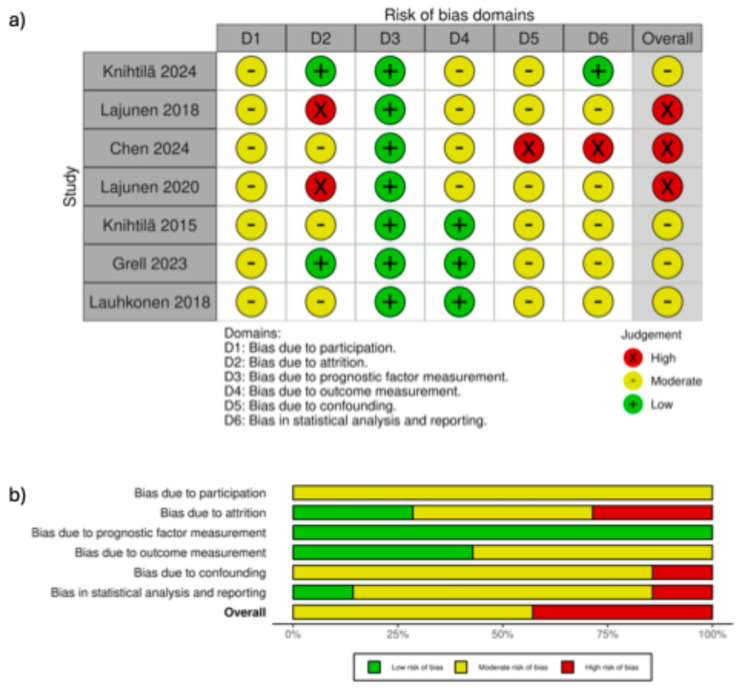
QUIPS risk of bias assessment of included studies. (**a**) shows domain-specific and overall risk of bias judgments for each included study [28,29,30,31,32,33,34]. (**b**) shows the percentage of studies judged to be at low, moderate, or high risk of bias for each QUIPS domain and overall.

Using QUIPS, no study was judged to be at low risk of bias across all domains. The most common concerns were related to study attrition, prognostic factor measurement, outcome measurement, and incomplete control for confounding. Overall, most studies were judged to be at moderate risk of bias, with greater concerns in studies affected by substantial attrition, selected analytic samples, or less robust outcome definitions. Detailed results are presented in Figure 2.

### 3.4. Preschool Oscillometry and Later Asthma-Related Outcomes

Across the included cohorts, abnormal oscillometric measurements obtained between 2 and 7 years of age were associated with later asthma-related outcomes, including subsequent asthma diagnosis, persistent symptoms, and medication use (Table 4). However, the studies were not interpreted as a single, uniform evidence base. Instead, they were considered according to follow-up horizon and clinical purpose, because a 1-year diagnostic or classification study addresses a different question from studies evaluating asthma persistence or lung function trajectories over several years. Accordingly, Chen et al. [31], which enrolled children aged 3–6 years, is presented as short-term diagnostic/classification evidence. In contrast, Knihtilä et al. [28], Lajunen et al. [30], and Lajunen et al. [32], with baseline oscillometry performed at ages 4, 4–7, and 3–7 years, respectively, are interpreted as longer-term prognostic evidence. Therefore, high AUC values from short-term studies were not treated as evidence of strong long-term predictive performance.

#### 3.4.1. Resistance at 5 Hz (R5)

R5 was the most consistently reported parameter. In the prospective multicenter study by Knihtilä et al. [28], higher R5 at age 4 was independently associated with active asthma at age 8 (β = 2.0; 95% CI 0.2–3.8; *p* = 0.029), with moderate discriminative ability (AUC 0.713). In the Finnish longitudinal cohort by Lajunen et al. [30], which included children aged 4–7 years with asthma-like symptoms, abnormal baseline R5 (z-score ≥ 1.645) was associated with asthma persistence into adolescence at ages 12–16 years, including higher odds of asthma symptoms (OR 9.9; 95% CI 2.9–34.4) and medication use (OR 9.2; 95% CI 2.7–31.7). In a subsequent analysis of asthmatic children aged 3–7 years from a related Finnish cohort [32], abnormal baseline R5 remained associated with asthma symptoms at 10-year follow-up, when participants were aged 12–16 years (OR 4.4; 95% CI 1.1–17.4). Separately, the short-term classification study by Chen et al. [31], which enrolled wheezing children aged 3–6 years, reported higher apparent classification accuracy for R5 after only 1 year of follow-up (AUC 0.91, sensitivity 0.89, specificity 0.84). Because of the short interval between baseline assessment and outcome ascertainment, this finding was interpreted as diagnostic/classification evidence rather than as evidence of strong long-term prognostic performance.

#### 3.4.2. Reactance at 5 Hz (X5) 

Reactance parameters also showed potential longitudinal relevance. In the Finnish cohort of asthmatic children aged 3–7 years [32], abnormal baseline X5 (z-score ≤ −1.645) was associated with asthma symptoms at 10-year follow-up, when participants were aged 12–16 years (OR 12.1; 95% CI 1.3–111.1). This finding suggests that early impairment in reactance-related respiratory mechanics may help identify children at increased risk of persistent disease.

In other cohorts, however, X5 was less consistently associated with later asthma-related outcomes than resistance-based measures [28,31]. Overall, these findings suggest that, although R5 provided the clearest and most recurrent individual signal across studies, reactance measures may offer complementary information on respiratory mechanics, ventilation heterogeneity, and asthma persistence, while not being specific for peripheral airway disease.

#### 3.4.3. Frequency-Dependent Resistance and Related Oscillometric Indices

Indices reflecting the frequency dependence of resistance, particularly R5-20, also showed potential prognostic relevance for later asthma-related outcomes. In the study by Knihtilä et al. [28], R5-20 demonstrated slightly higher discriminative performance than R5 alone (AUC 0.726 vs. 0.713), suggesting that altered frequency-dependent respiratory mechanics may contribute to later asthma risk. Similarly, the Finnish cohort by Lajunen et al. [30] reported associations between several oscillometric indices, including R5-20 and other measures of frequency-dependent resistance, and later airflow limitation.

However, thresholds, parameter expression, covariate adjustment, and study populations differed across studies. Therefore, the available evidence does not currently support recommending any single frequency-dependent or reactance-derived metric for routine clinical use.

#### 3.4.4. Overall Interpretation

Overall certainty for later asthma-related outcomes was judged to be low. Although the direction of association was generally consistent, confidence in the evidence was limited by the small number of studies, moderate-to-high risk of bias in several cohorts, imprecision of some estimates, heterogeneity in oscillometric metrics and outcome definitions, and potential non-independence of some Finnish asthma reports, particularly the apparent overlap between Lajunen 2018 [30] and Lajunen 2020 [32]. Nevertheless, abnormal preschool oscillometric parameters were directionally associated with later asthma-related outcomes, with resistance-based measures showing the clearest recurring signal. Frequency-dependent resistance and reactance indices may provide complementary information, but current evidence is insufficient to identify a preferred metric or clinically applicable threshold.

The longer-term prognostic interpretation is based mainly on cohorts with 4- to 10-year follow-up. The 1-year study was retained because it was relevant to preschool oscillometry and subsequent asthma classification; however, its high AUC values were interpreted separately and were not considered evidence of equivalent long-term predictive accuracy.

**Figure 3:**
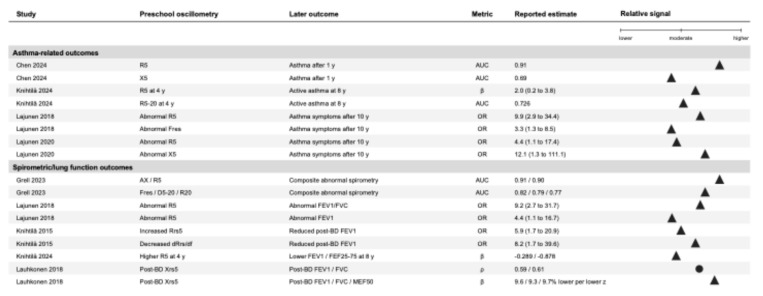
Semi-quantitative evidence map of preschool oscillometry associations with later asthma-related and spirometric/lung function outcomes. The upper section summarizes asthma-related outcomes, and the lower section summarizes spirometric/lung function outcomes, including FEV_1_, FEV_1_/FVC, FEF_25–75_, FVC, and MEF_50_, where reported. Each row identifies the study, oscillometric parameter, later outcome, metric, reported estimate, and approximate relative signal strength. Because studies differed in populations, oscillometric indices, thresholds, follow-up periods, and effect measures, estimates were not pooled. Marker position provides an approximate visual summary of relative signal strength on the native reported scale. Triangles indicate adverse-direction associations, in which higher resistance, frequency dependence, reactance area, or more negative reactance was linked with worse outcomes. Circles indicate favorable-direction reactance associations, where higher or less negative Xrs5 was associated with better spirometric values. AUC, OR, β, and ρ are displayed on their native reported scales; heterogeneity precluded meta-analysis [28,29,30,31,32,33,34].

To address heterogeneity in reported estimates, Figure 3 presents a semi-quantitative harvest/effect-size evidence map rather than a formal forest plot. The figure separates asthma-related outcomes from spirometric/lung function outcomes and specifies the oscillometric parameter, later outcome, metric, and reported estimate for each association. This format clarifies whether the outcome was asthma diagnosis, symptoms, active asthma, or a specific spirometric measure, such as FEV_1_, FVC, FEV_1_/FVC, or FEF_25–75_. Because studies differed in populations, indices, thresholds, follow-up periods, and effect measures, estimates were not pooled; marker position provides an approximate visual summary of relative signal strength on the native reported scale.

### 3.5. Preschool Oscillometry and Long-Term Lung Function

In most longitudinal cohorts (Table 5), oscillometric abnormalities measured between 2 and 7 years of age were associated with reduced spirometric indices at school age or adolescence. Although studies differed in population characteristics, the overall direction of association was generally consistent. Higher airway resistance and impaired reactance during baseline testing at ages 2–7 years were linked to later airflow limitation.

R5/Rrs5 was the most consistently evaluated parameter. In the multicenter cohort by Knihtilä et al. [28], higher R5 at age 4 years was associated with lower FEV_1_ and FEF_25–75_ between ages 5 and 8 years, suggesting that early resistance abnormalities may track with subsequent airflow limitation. The Chilean cohort of children with persistent asthma [29] was interpreted separately as a shorter, 3-year school-age classification study. In this study, IOS performed at ages 3–5 years was followed by spirometry at ages 6–8 years, and preschool R5, R20, AX, and D5-20 were associated with abnormal spirometry at school age, with moderate discriminative accuracy (AUC >0.7). R5 and AX showed the highest apparent predictive performance within that cohort. These findings suggest that increased total airway resistance at ages 3–5 years may reflect functional abnormalities that persist into later childhood.

Longer follow-up studies extending into adolescence further support this pattern. In a Finnish cohort of asthmatic children with IOS performed at ages 2–7 years and spirometry at ages 12–18 years [33], increased baseline Rrs5 and decreased dRrs/df were associated with reduced post-bronchodilator FEV_1_ in adolescence, with ORs of 5.9 and 8.2, respectively. The persistence of reduced lung function after bronchodilator administration suggests that early oscillometric abnormalities may reflect more persistent airway dysfunction rather than purely reversible bronchoconstriction.

Reactance measures also showed longitudinal relevance. In children with a history of severe bronchiolitis [34], IOS performed at ages 5–7 years showed that Xrs5 correlated with spirometric outcomes at ages 11–13 years. Post-bronchodilator Xrs5 demonstrated moderate-to-strong correlations with post-bronchodilator FEV_1_ and FVC (ρ = 0.59 and 0.61, respectively). In addition, each unit decrease in baseline post-bronchodilator Xrs5 z-score was associated with approximately 9–10% lower spirometric indices in adolescence. These findings suggest that reactance-related mechanics and elastic properties may be relevant to long-term lung function trajectories, although they should not be interpreted as anatomically specific evidence of peripheral airway disease.

In children aged 4–7 years with asthma-like symptoms, abnormal baseline R5 was also associated with a reduced FEV_1_/FVC ratio at ages 12–16 years (OR 9.2; 95% CI 2.7–31.7) [30], reinforcing the association between early resistance abnormalities and later airflow obstruction. In contrast, analyses focusing primarily on airway hyperresponsiveness did not consistently demonstrate independent associations between preschool oscillometric parameters and adolescent spirometry [32], suggesting that the predictive value of oscillometry may vary according to cohort characteristics, outcome definition, and statistical modeling approach.

Overall certainty for later lung function impairment was judged to be low. Although the direction of association was generally consistent across most cohorts, confidence in the evidence was limited by heterogeneity in study populations, spirometric endpoints, oscillometric expression, and follow-up duration, as well as by moderate risk of bias, limited sample sizes, partial cohort overlap, and the observational design of the included studies. Within these limitations, resistance-based measures showed the clearest recurring association with later airflow limitation, whereas reactance and frequency-dependent indices appeared to provide complementary information on respiratory mechanics, including ventilation heterogeneity, elastic properties, or possible peripheral airway involvement. These relationships are summarized in Figure 3, which visually separates asthma-related outcomes from spirometric/lung function outcomes and displays the reported estimates on their native scales. Overall, the available evidence suggests that early oscillometric abnormalities may track longitudinally and may be associated with later lung function impairment in childhood and adolescence.

## 4. Discussion

This systematic review evaluated whether oscillometric measurements obtained between 2 and 7 years of age are associated with later asthma-related outcomes and long-term lung function impairment. Across the included longitudinal cohorts, abnormal oscillometry, particularly elevated resistance-based indices such as R5, was generally associated with later asthma-related outcomes and lower subsequent lung function. However, the included cohorts mainly comprised children with wheezing, asthma-like symptoms, persistent asthma, prior hospitalization for bronchiolitis, or other high-risk asthma backgrounds, rather than unselected apparently healthy children. Therefore, these findings suggest that preschool oscillometry may provide useful complementary physiological information for risk assessment in symptomatic or high-risk preschool-aged children and should not be extrapolated to routine screening of apparently healthy populations.

R5 was the most consistent individual parameter across studies. Longer-term cohorts reported higher odds of later asthma symptoms or medication use among children with abnormal R5 measured between ages 3 and 7 years [30,32], and the multicenter VDAART cohort linked higher age-4 R5 with active asthma and lower spirometric indices at school age [28]. These findings support the use of resistance-based oscillometric information as an adjunct to clinical assessment, while also recognizing that some Finnish asthma reports were related and should not be interpreted as fully independent replications.

The reviewed studies also differed in clinical purpose and follow-up interval. The 1-year study by Chen et al. [31] is best interpreted as short-term diagnostic/classification evidence, whereas studies with 4- to 10-year follow-up more directly address asthma persistence and lung-function trajectories. This distinction is clinically important because high AUC values from short-term classification studies should not be extrapolated to long-term prediction of persistent asthma.

Beyond asthma-related outcomes, oscillometric abnormalities measured between ages 2 and 7 years were also associated with later lung function impairment, including lower FEV_1_, FEV_1_/FVC, FEF_25–75,_ FVC, or MEF_50_ in some cohorts [28,30,33,34]. Reactance and frequency-dependent resistance indices appeared to provide complementary information on respiratory mechanics, but these parameters were less consistently reported, physiologically nonspecific, and expressed using non-uniform metrics. Therefore, current evidence does not support a universal oscillometric threshold or a single preferred reactance-derived or frequency-dependent index for clinical implementation.

From a clinical perspective, oscillometry may be most useful when integrated into a broader risk-assessment framework for symptomatic or high-risk preschool-aged children. Abnormal or persistently elevated R5, and potentially abnormalities in R5-20, X5, AX, or Fres should be interpreted alongside clinical features such as wheezing pattern, exacerbation frequency, interval symptoms, bronchodilator response, atopy, eczema, family history, environmental exposures, fractional exhaled nitric oxide (FeNO) or other available biomarkers, and treatment response. Within this context, abnormal oscillometry may support closer follow-up, repeat objective assessment, optimization of adherence and trigger control, or referral to pediatric pulmonology or allergy services. However, it should not be used as a stand-alone screening test or as the sole basis for diagnosis or treatment escalation.

This review has several limitations. The number of eligible longitudinal studies was small, and most were judged to be at moderate or high risk of bias, mainly because of attrition and incomplete control for confounding. In addition, some Finnish asthma reports were not fully independent, which limits their interpretation as separate replications. Although major databases were searched, the final evidence base remained limited, and the restriction to English-language publications may have led to the exclusion of relevant studies. Most included cohorts consisted of symptomatic or high-risk children, limiting the generalizability of the findings to broader preschool populations. Methodological heterogeneity was also substantial, reflecting differences in baseline phenotypes, devices, signal type, bronchodilator conditions, reference equations, parameter expression, outcome definitions, follow-up duration, and reporting approaches. These differences limit direct comparability and may partly explain why no single transferable threshold can currently be recommended. Moreover, several cohorts were also conducted before publication of the 2020 European Respiratory Society (ERS) Technical Standards for Respiratory Oscillometry, which provide recommendations on equipment performance, calibration and verification, patient preparation, acceptability and repeatability criteria, quality control, and reporting [21]. More recent pediatric standardization initiatives, including the STAN PNEUMO oscillometry statement, further emphasize the need for harmonized methodology, interpretation, and local implementation based on ERS recommendations [36].

Standardization is therefore essential before preschool oscillometry can be translated into prognostic practice. Future studies should apply harmonized IOS/FOT protocols aligned with ERS technical standards, and should transparently report device type, calibration and verification procedures, patient posture, cheek support, nose clip use, recording duration, number of acceptable trials, artifact rejection procedures, within-session repeatability, and bronchodilator status [21]. These details are particularly important in preschool children, in whom cooperation, tidal breathing variability, upper airway shunting, and quality control decisions can substantially influence resistance and reactance measurements. Reference values and thresholds also require harmonization. Ideally, oscillometric results should be reported as z-scores derived from appropriate age-, height-, sex-, and population-adjusted reference equations, with absolute values and percent-predicted values provided when helpful for clinical interpretation. Prespecified age-adjusted thresholds should be externally validated rather than derived post hoc within small cohorts. Until such standardization is achieved, R5 appears to be the most reproducible individual marker across longitudinal studies, whereas R5-20, X5, AX, Fres, and dRrs/df should be regarded as complementary, physiologically non-specific indices rather than interchangeable or universally applicable prognostic thresholds.

Future studies should move beyond associations and assess whether oscillometry improves risk prediction beyond established clinical predictors and biomarkers. Large multicenter cohorts should use ERS-aligned protocols, prespecified thresholds, harmonized asthma and lung-function outcomes, and transparent reporting of device type, quality-control criteria, bronchodilator status, reference equations, and parameter expression. Clinical-impact studies are also needed to determine whether oscillometry-guided monitoring or management improves asthma control, exacerbation risk, treatment decisions, or long-term lung-function trajectories.

Overall, the evidence supports oscillometry as a feasible adjunctive tool that may add objective physiological information to asthma-risk assessment in selected preschool-aged children. The most consistent signal relates to resistance-based measures, particularly R5, while reactance and frequency-dependent indices may provide additional context but require further standardization before routine prognostic use.

## 5. Conclusions

This systematic review found that oscillometric abnormalities measured during the preschool-age range, particularly elevated R5, were associated with later asthma-related outcomes and subsequent lung function impairment. These associations were observed mainly in children with wheezing, asthma-like symptoms, prior hospitalization for bronchiolitis, or other high-risk respiratory phenotypes. Resistance-based indices showed the most consistent longitudinal signal, whereas reactance and frequency-dependent resistance measures appeared to provide complementary but less standardized information.

Overall, preschool oscillometry may add objective physiological information to clinical assessment and risk stratification in symptomatic or clinically high-risk preschool-aged children, particularly when elevated R5 is present. These findings support the use of oscillometry as an adjunctive tool within a broader clinical framework, while further standardized prospective studies are needed before routine stand-alone prognostic use can be recommended.

## Figures and Tables

**Table 1:** Practical interpretation of commonly reported oscillometry indices.

Domain	Index	What It Mainly Represents	Unfavorable Direction/Clinical Note
Resistance	R5	Total respiratory system resistance, including central and peripheral airway contributions.	Higher values suggest increased airway narrowing or obstruction; R5 is the most established individual IOS/FOT marker in pediatric studies.
Resistance	R20	Resistance at a higher frequency, relatively more influenced by the central/proximal airways.	Higher values suggest increased airway resistance, mainly reflecting larger/proximal airway contributions.
Frequency-dependent resistance	R5-20; dRrs/df	Variation in resistance across frequencies; used as an indirect, nonspecific signal of frequency-dependent mechanics, ventilation heterogeneity, or possible peripheral airway involvement.	Higher R5-20 is generally unfavorable. A more abnormal slope may suggest greater frequency dependence, but dRrs/df is less standardized, not routinely used clinically, and not anatomically specific.
Reactance	X5	Elastic and inertial properties of the respiratory system at low frequency.	More negative values may suggest reduced compliance, altered elastic properties, ventilation heterogeneity, or possible peripheral airway obstruction.
Reactance-derived	AX	Cumulative reactance abnormality between 5 Hz and Fres.	Higher values suggest greater reactance abnormality and may be compatible with peripheral airway dysfunction or reduced compliance.
Reactance-derived	Fres	Frequency at which reactance crosses zero.	Higher values generally suggest worse mechanical abnormality and possible peripheral airway involvement but are not anatomically specific.

IOS, impulse oscillometry; FOT, forced oscillation technique; R, resistance; X, reactance; AX, area of reactance; Fres, resonant frequency; dRrs/df, slope of resistance versus frequency.

**Table 2:** PICOTS framework for the review question and eligibility concept.

PICOTS Component	Description
**P**—Population	Preschool-aged children or early-childhood populations, generally aged ≤6 years, including children with wheezing, asthma-like symptoms, persistent asthma, or children at risk of later asthma. Studies with measurements obtained slightly beyond age 6 years could also be included if the population and clinical question remained focused on preschool wheezing and later asthma risk. In the final included studies, baseline oscillometry was performed between the ages of 2 and 7 years.
**I**—Intervention, Exposure, or Prognostic Factor	Oscillometric assessment performed during the preschool age of the included studies (2–7 years), including impulse oscillometry (IOS) or forced oscillation technique (FOT) parameters such as resistance at 5 Hz (R5), resistance at 20 Hz (R20), frequency dependence of resistance (R5-20), reactance at 5 Hz (X5), area of reactance (AX), and resonant frequency (Fres).
**C**—Comparison (if applicable)	Children with normal versus abnormal oscillometric findings, or comparisons across oscillometric values, thresholds, z-scores, or parameter levels.
**O**—Outcome	Later asthma-related outcomes, including physician-diagnosed asthma, active asthma, asthma symptoms, and/or asthma medication use, as well as subsequent lung function impairment, such as reduced FEV_1_, reduced FEV_1_/FVC, or persistent airflow obstruction.
**T**—Time	Longitudinal follow-up from preschool or early childhood oscillometric assessment to school age, later childhood, or adolescence.
**S**—Study Design	Longitudinal observational studies, including prospective and retrospective cohort studies.

**Review question:** Are abnormal oscillometric measurements obtained at preschool age (2–7 years in the included studies) associated with later asthma-related outcomes or long-term lung function impairment?

**Table 3:** Characteristics of the included studies.

Study	Country	Design	Population	Baseline Age at Oscillometry	Oscillometry Parameters	Follow-Up	Age at Outcome Assessment	Primary Outcome(s)
Knihtilä 2024 [28]	USA	Secondary analysis of prospective multicenter birth cohort (VDAART)	220 children from a high-risk asthma cohort	4, 5, and 6 years; main analysis at 4 years	R5, R5-20, X5, AX	4 years	8 years for active asthma; 5–8 years for repeated spirometry	Active asthma, repeated spirometry
Chen 2024 [31]	China	Prospective longitudinal cohort	213 children with wheezing	3–6 years; median 53 months (4.4 years)	R5, X5, R20, Fres, R5-R20	1 year	Approximately 4–7 years	Asthma versus non-asthma classification according to mAPI-based criteria
Grell 2023 [29]	Chile	Non-randomized prospective cohort	66 children with persistent asthma	3–5 years; mean 4.9 years	R5, R20, X5, Fres, AX, D5-20	3 years	6–8 years; mean 7.9 years	Abnormal spirometry and bronchodilator response
Lajunen 2018 [30]	Finland	Prospective longitudinal cohort	255 children with asthma-like symptoms; 121 attended teenage follow-up	4–7 years; median 5 years	R5, R20, R5-20, dR/df, X5, X10, Fres	10 years	12–16 years	Asthma symptoms, asthma medication use, and abnormal FEV_1_, FEV_1_/FVC
Lajunen 2020 [32]	Finland	Prospective longitudinal cohort	105 asthmatic children; 64 attended follow-up	3–7 years	R5, X5	10 years	12–16 years	Active asthma symptoms and airway hyper-responsiveness
Knihtilä 2015 [33]	Finland	Retrospective longitudinal cohort	154 children with asthma	2–7 years	Rrs5, Rrs20, dRrs/df, Xrs5, Xrs10, Fres	At least 5 years; median 9.1 years	12–18 years; median 13.5 years	Spirometry, especially post-bronchodilator FEV_1_, FEV_1_/FVC and MEF50
Lauhkonen 2018 [34]	Finland	Prospective longitudinal cohort	64 children hospitalized for bronchiolitis	5–7 years; mean 6.3 years	Rrs5, Xrs5	~5.1 years	11–13 years; mean 11.4 years	Flow-volume spirometry outcomes

IOS, impulse oscillometry; VDAART, Vitamin D Antenatal Asthma Reduction Trial; R5, resistance at 5 Hz; R20, resistance at 20 Hz; R5-20, difference between resistance at 5 and 20 Hz; dR/df and dRrs/df, slope of resistance versus frequency; X5, reactance at 5 Hz; AX, area of reactance; Fres, resonant frequency; D5-20, difference between resistance at 5 and 20 Hz; Rrs5, respiratory system resistance at 5 Hz; Rrs20, respiratory system resistance at 20 Hz; Xrs5, respiratory system reactance at 5 Hz; Xrs10, respiratory system reactance at 10 Hz; mAPI, modified Asthma Predictive Index; FEV_1_, forced expiratory volume in 1 s; FEV_1_/FVC, ratio of forced expiratory volume in 1 s to forced vital capacity; MEF_50_, maximal expiratory flow at 50% of forced vital capacity. Cohort overlap note: Lajunen 2018 and Lajunen 2020 appear to arise from the same Helsinki longitudinal asthma cohort, with Lajunen 2020 likely representing a related sub-cohort or follow-up analysis. Knihtilä 2015 was also conducted in the Helsinki pediatric asthma setting and is therefore institutionally related, although direct participant overlap with the Lajunen cohorts is not clearly documented. Lauhkonen 2018 was a separate Finnish post-bronchiolitis cohort, and the U.S., Chinese, and Chilean studies were independent cohorts. In the narrative synthesis, ‘preschool-aged’ refers to the age range represented by the included baseline oscillometry assessments (2–7 years), unless a narrower study-specific age range is stated.

**Table 4:** Preschool oscillometry and later asthma-related outcomes, separated by evidence type.

Evidence Type	Study	IOS Metric	Threshold/Scale	Outcome Definition	Key Result for Later Asthma-Related Outcome
**Short-term diagnostic/classification evidence (1-year follow-up)**	Chen 2024 [31]	R5	Cutoff 119.45% predicted	Asthma diagnosis after 1-year follow-up	AUC 0.91; sensitivity 0.89; specificity 0.84
	Chen 2024 [31]	X5	Cutoff 145.95% predicted	Asthma diagnosis after 1-year follow-up	AUC 0.69; sensitivity 0.99; specificity 0.33
**Longer-term prognostic evidence (4- to 10-year follow-up)**	Knihtilä 2024 [28]	R5	Continuous age-4 R5	Active asthma at age 8 years	Higher R5 was associated with active asthma at age 8 (β = 2.0, 95% CI 0.2–3.8; *p* = 0.029); ROC AUC 0.713
	Knihtilä 2024 [28]	R5-20	Continuous age-4 R5-20	Active asthma at age 8 years	Discrimination slightly higher than R5 alone (ROC AUC 0.726)
	Lajunen 2018 [30]	Abnormal R5	z-score ≥ 1.645	Asthma symptoms and asthma medication use in adolescence	Symptoms: OR 9.9 (95% CI 2.9–34.4); medication use: OR 9.2 (95% CI 2.7–31.7)
	Lajunen 2018 [30]	Abnormal Fres	z-score ≥ 1.645	Asthma symptoms and asthma medication use in adolescence	Symptoms: OR 3.3 (95% CI 1.3–8.5); medication use: OR 3.7 (95% CI 1.4–9.3)
	Lajunen 2020 [32]	Abnormal R5	z-score ≥ 1.645	Asthma symptoms at 10-year follow-up	OR 4.4 (95% CI 1.1–17.4)
	Lajunen 2020 [32]	Abnormal X5	z-score ≤ −1.645	Asthma symptoms at 10-year follow-up	OR 12.1 (95% CI 1.3–111.1)

IOS, impulse oscillometry; R5, resistance at 5 Hz; R5-20, difference between resistance at 5 and 20 Hz; X5, reactance at 5 Hz; Fres, resonant frequency; AUC, area under the receiver operating characteristic curve; ROC, receiver operating characteristic; OR, odds ratio; CI, confidence interval. Short-term diagnostic/classification evidence and longer-term prognostic evidence are presented separately because they address different clinical questions. AUC values from 1-year classification studies should not be interpreted as evidence of long-term predictive performance.

**Table 5:** Preschool oscillometry and later lung function impairment.

Evidence Type	Study	IOS Metric(s)	Later Lung Function Outcome	Key Finding
**Short-term school-age lung function evidence**	Knihtilä 2024 [28]	R5	Repeated spirometry from ages 5–8 years	Higher age-4 R5 was associated with lower lung function from ages 5–8; for FEV_1_ at age 8, β = −0.3 (95% CI −0.5 to −0.1; *p* < 0.001)
	Grell 2023 [29]	R5, R20, Fres, AX, D5-20	Abnormal spirometry at school age	R5 AUC 0.90, AX AUC 0.91, Fres AUC 0.82, D5-20 AUC 0.79, R20 AUC 0.77 for abnormal spirometry; AX, D5-20 and R5 had the highest LR+
**Long-term adolescent lung function evidence**	Lajunen 2018 [30]	Abnormal R5/abnormal Fres	Abnormal adolescent spirometry	Abnormal preschool R5 was associated with abnormal FEV_1_ OR 4.4 (1.1 to 16.7) and abnormal FEV_1/_FVC OR 9.2 (2.7 to 31.7); abnormal Fres was associated with abnormal FEV_1_/FVC OR 5.9 (2.2 to 15.8)
	Knihtilä 2015 [33]	Rrs5, dRrs/df, Fres	Post-bronchodilator adolescent spirometry	Increased Rrs5 was associated with reduced post-BD FEV_1_ (OR 5.9, 95% CI 1.7–20.9) and reduced FEV_1_/FVC (OR 4.2, 95% CI 1.5–11.8); decreased dRrs/df was associated with reduced post-BD FEV_1_ (OR 8.2, 95% CI 1.7–39.6)
	Lajunen 2020 [32]	R5 and X5	Adolescent spirometry/AHR-focused follow-up	No clear independent longitudinal spirometric signal highlighted; study mainly linked preschool IOS to later asthma symptoms and AHR
	Lauhkonen 2018 [34]	Baseline and post-BD Xrs5; baseline and post-BD Rrs5	Early adolescent FVS	Post-BD Xrs5 correlated strongly with post-BD FEV_1_ (rho 0.59) and FVC (rho 0.61); each 1-unit decrease in preschool post-BD Xrs5 z-score predicted about 9.6% lower post-BD FEV_1_, 9.3% lower FVC, and 9.7% lower MEF50

IOS, impulse oscillometry; R5, resistance at 5 Hz; R20, resistance at 20 Hz; Fres, resonant frequency; AX, area of reactance; D5–20, difference between resistance at 5 and 20 Hz; Rrs5, respiratory system resistance at 5 Hz; dRrs/df, slope of resistance versus frequency; X5, reactance at 5 Hz; Xrs5, respiratory system reactance at 5 Hz; BD, bronchodilator; FEV_1_, forced expiratory volume in 1 s; FVC, forced vital capacity; FEV_1_/FVC, ratio of forced expiratory volume in 1 s to forced vital capacity; FVS, flow-volume spirometry; MEF_50_, maximal expiratory flow at 50% of forced vital capacity; AHR, airway hyper-responsiveness; AUC, area under the receiver operating characteristic curve; LR+, positive likelihood ratio; OR, odds ratio; CI, confidence interval.

## Data Availability

No new data were created in this study. Data supporting the findings of this systematic review were derived from previously published articles, which are cited in the reference list of this manuscript.

## Supplementary Materials

The following supporting information can be downloaded at: https://www.mdpi.com/article/10.3390/children13060756/s1, Table S1: PRISMA 2020 Checklist.

## Abbreviations

The following abbreviations are used in this manuscript:

R5	Resistance at 5 Hz
FEV_1_	Forced expiratory volume in one second
FVC	Forced vital capacity
FEV_1_/FVC	Ratio of forced expiratory volume in one second to forced vital capacity
IOS	Impulse Oscillometry
FOT	Forced Oscillation Technique
R20	Resistance at 20 Hz
R5-20	Difference between resistance at 5 Hz and 20 Hz
dRrs/df	slope of resistance versus frequency
X5	Reactance at 5 Hz
AX	Area of reactance
Fres	Resonant frequency
PRISMA	Preferred Reporting Items for Systematic Reviews and Meta-Analyses
PROSPERO	International Prospective Register of Systematic Reviews
PICOTS	Population, Intervention/Exposure, Comparison, Outcome, Timing, and Study design
QUIPS	Quality In Prognosis Studies
OR	Odds ratio
AUC	Area under the curve
CI	Confidence interval
VDAART	Vitamin D Antenatal Asthma Reduction Trial
D5-20	Difference between resistance at 5 Hz and 20 Hz
mAPI	Modified Asthma Predictive Index
dR/df	Slope of resistance versus frequency
X10	Reactance at 10 Hz
Rrs5	Respiratory system resistance at 5 Hz
Rrs20	Respiratory system resistance at 20 Hz
Xrs5	Respiratory system reactance at 5 Hz
Xrs10	Respiratory system reactance at 10 Hz
MEF_50_	Maximal expiratory flow at 50% of forced vital capacity
ROC	Receiver operating characteristics
FEF_25–75_	Forced expiratory flow at 25–75% of vital capacity
β	Beta coefficient
ρ	Correlation coefficient
BD	Bronchodilator
LR+	Positive likelihood ratio
AHR	Airway hyper-responsiveness
FVS	Flow-volume spirometry
FeNO	Fractional exhaled nitric oxide
ERS	European Respiratory Society
STAN PNEUMO	Pediatric pulmonology oscillometry standardization statement

## Appendix A

**Table A1:** Search concepts, free-text keywords, and controlled vocabulary used across databases.

Concept	Free-Text Keywords	Controlled Vocabulary/Subject Headings
**Oscillometry**	impulse oscillometry; forced oscillation technique; forced oscillation techniques; oscillometry; airway oscillometry; respiratory oscillometry; IOS; FOT	Oscillometry
**Preschool children**	preschool*; pre-school; toddler*; young child*; early childhood	Child, Preschool
**Asthma/wheezing**	asthma*; wheez*; recurrent wheezing; recurrent wheez*	Asthma
**Longitudinal outcomes/prognosis**	longitudinal; longitudinal*; follow-up; cohort; cohort*; prospective; predict*; risk; disease development; disease progression	Disease Progression; Longitudinal Studies

Abbreviations: IOS, impulse oscillometry; FOT, forced oscillation technique. The asterisk (*) indicates truncation/wildcard searching and was used to retrieve words with different endings or spelling variants; for example, asthma retrieves asthma and asthmatic, wheez* retrieves wheeze, wheezing, and wheezy, and toddler* retrieves toddler and toddlers. Search terms were adapted to the syntax and indexing system of each database. Free-text terms were combined with controlled vocabulary where available, using Boolean operators (AND, OR). Truncation symbols and phrase searching were applied according to individual database requirements. The complete database-specific search strategies are provided below.

**Table A2:** Detailed search strategy for each database.

Database	Date Last Searched	Search Strategy
**PubMed**	28 February 2026	(“Oscillometry” [Mesh] OR “impulse oscillometry” OR “forced oscillation technique” OR FOT OR IOS OR oscillometry) AND (“Child, Preschool” [Mesh] OR preschool* OR “pre-school” OR toddler* OR “young child*” OR “early childhood”) AND (“Asthma” [Mesh] OR asthma* OR wheez* OR “recurrent wheezing”)
		AND (“Disease Progression” [Mesh] OR “Longitudinal Studies” [Mesh] OR longitudinal* OR follow-up OR cohort* OR prospective OR predict* OR risk OR “disease development”)
**Scopus**	28 February 2026	(TITLE-ABS-KEY (“impulse oscillometry” OR “forced oscillation technique” OR IOS OR FOT OR oscillometry)) AND (TITLE-ABS-KEY (preschool* OR “pre-school” OR toddler* OR “young child*” OR “early childhood”)) AND (TITLE-ABS-KEY (asthma* OR wheez* OR “recurrent wheezing”)) AND (TITLE-ABS-KEY (longitudinal OR “follow-up” OR cohort OR prospective OR predict* OR risk OR “disease progression”))
**MEDLINE (via EBSCOhost)**	28 February 2026	(MH “Oscillometry+” OR TI “impulse oscillometry” OR AB “impulse oscillometry” OR TI “forced oscillation technique” OR AB “forced oscillation technique” OR TI “forced oscillation techniques” OR AB “forced oscillation techniques” OR TI oscillometr* OR AB oscillometr* OR TI “airway oscillometry” OR AB “airway oscillometry” OR TI “respiratory oscillometry” OR AB “respiratory oscillometry” OR (TI IOS AND TI respir*) OR (AB IOS AND AB respir*) OR (TI FOT AND TI respir*) OR (AB FOT AND AB respir*)) AND (MH “Child, Preschool+” OR TI preschool* OR AB preschool* OR TI pre-school OR AB pre-school OR TI toddler* OR AB toddler* OR TI “young child*” OR AB “young child*” OR TI “early childhood” OR AB “early childhood”) AND (MH “Asthma+” OR TI asthma* OR AB asthma* OR TI wheez* OR AB wheez* OR TI “recurrent wheez*” OR AB “recurrent wheez*”) AND (MH “Disease Progression+” OR MH “Longitudinal Studies+” OR TI longitudinal* OR AB longitudinal* OR TI follow-up OR AB follow-up OR TI cohort* OR AB cohort* OR TI prospective OR AB prospective OR TI predict* OR AB predict* OR TI prognos* OR AB prognos* OR TI risk OR AB risk OR TI “disease development” OR AB “disease development” OR TI “disease progression” OR AB “disease progression”)

Abbreviations: IOS, impulse oscillometry; FOT, forced oscillation technique; MeSH, Medical Subject Headings; TI, title; AB, abstract; MH, subject heading. The asterisk (*) indicates truncation/wildcard searching and was used to retrieve words with different endings or spelling variants, according to the syntax of each database; for example, asthma retrieves asthma and asthmatic, wheez* retrieves wheeze, wheezing, and wheezy, and predict* retrieves predict, predicts, predicted, and prediction. Quotation marks were used for phrase searching where appropriate. Search terms were adapted to the syntax and indexing system of each database.
